# A novel tetramethylnaphthalene derivative synergistically inhibits HTLV-1-infected cell proliferation in combination with cepharanthine

**DOI:** 10.1186/1742-4690-8-S1-A64

**Published:** 2011-06-06

**Authors:** Masaaki Toyama, Takayuki Hamasaki, Tomofumi Uto, Hiroshi Aoyama, Mika Okamoto, Yuichi Hashimoto, Masanori Baba

**Affiliations:** 1 Center for Chronic Viral Diseases, Kagoshima University, Kagoshima 890-8544, Japan; 2 Institute of Molecular and Cellular Biosciences, The University of Tokyo, Tokyo 113-0032, Japan

## Background

We have previously found that the novel tetramethylnaphthalene derivative TMNAA selectively inhibits the proliferation of HTLV-1-infected T-cell lines but not HTLV-1-uninfected T-cell lines. Although its target molecule is still unknown, TMNAA did not affect NF-κB activity. Therefore, we further examined the anti-proliferative activity of TMNAA against various T-cell lines in combination with cepharanthine (CEP), which is known to inhibit NF-κB.

## Materials and methods

HTLV-1-infected and uninfected T-cell lines were cultured in the presence of various concentrations of TMNAA and CEP, and their proliferation and viability were determined by a tetrazolium dye method. The mode of cell death was also examined by flow cytometry and Western blot analysis.

## Results

The 50% inhibitory concentrations (IC50s) of TMNAA and CEP for the ATL cell line (S1T) were 1.65 ± 0.03 and 1.97 ± 0.29 µM, respectively. On the other hand, the IC50 of TMNAA and CEP combination resulted in 0.93 ± 0.13 µM, indicating that the combination synergistically inhibited the proliferation of S1T cells. Such synergism was observed for another infected cell line (MT-2) but not for HTLV-1-uninfected cell lines. Moreover, TMNAA did not induce apoptosis of S1T cells, but CEP did. Interestingly, TMNAA significantly enhanced the CEP-induced apoptosis of S1T and MT-2 cells.

## Conclusions

The combination of TMNAA and CEP selectively inhibits the proliferation of HTLV-1-infected cell lines through the induction of apoptosis. Therefore, TMNAA and CEP may have potential for chemotherapy of ATL.

